# Syndrome des antisynthétases : sept observations du service de médecine interne du CHU de Libreville, Gabon

**DOI:** 10.48327/mtsi.v5i2.2025.711

**Published:** 2025-06-25

**Authors:** Josaphat IBA BA, Annick MFOUMOU, Ingrid NSENG-NSENG ONDO, Ulrich Davy KOMBILA, Jean-Bruno BOGUIKOUMA

**Affiliations:** 1 Service de médecine interne, CHU de Libreville, BP 2228, Libreville, Gabon; 2 Service de rhumatologie, CHU de Libreville, BP 2228, Libreville, Gabon

**Keywords:** Syndrome des antisynthétases, Main de mécanicien, Pneumopathie interstitielle diffuse, Polyarthrite, Syndrome de Raynaud, Gabon, Afrique subsaharienne, Antisynthetases syndrome, Mechanic's hands, Diffuse interstitial pneumopathy, Polyarthritis, Raynaud's syndrome, Sub-Saharan AfricaIntroduction

## Abstract

**Introduction:**

Le syndrome des antisynthétases (SAS) est un sous-groupe de myopathie inflammatoire, dans lequel la myosite se retrouve associée à une polyarthrite, une pneumopathie interstitielle, un phénomène de Raynaud, des lésions cutanées de type de « mains de mécanicien », et une positivité d'anticorps anti-aminoacyl-transfer RNA (tRNA) synthétase qui conforte le diagnostic. Nous rapportons sept observations gabonaises.

**Matériel et méthodes:**

Il s'agit d'une étude rétrospective et descriptive, réalisée dans le service de médecine interne du CHU de Libreville, recensant sur une période allant du 1^er^ janvier 1984 au 31 décembre 2023 tous les patients avec SAS documenté, dans le but d'en préciser les différentes caractéristiques.

**Résultats:**

Sept patientes de 42,5 ans d’âge moyen ont été retrouvées. Dans les signes cardinaux habituels du SAS prédominaient une atteinte musculaire (n = 7), pulmonaire (n = 5) avec pneumopathie interstitielle diffuse (n = 5), compliquée de dyspnée de stade 2 (n = 3) à 3 (n = 2), cutanée à type de « mains de mécanicien » (n = 4), et positivité des anticorps antisynthétases Jo-1 (n = 2), PL7 (n = 1) et PL12 (n = 4).

**Discussion:**

Le SAS constitue un véritable défi pour le clinicien d'Afrique subsaharienne du fait de la pneumopathie interstitielle diffuse qui accompagne l'atteinte pulmonaire.

**Conclusion:**

Notre étude conforte la prédominance d'anticorps anti PL12 et le caractère tardif du diagnostic de cette affection.

## Abstract (en)

**Introduction:**

Antisynthetases syndrome (AS) is a subgroup of inflammatory myopathy where myositis is associated to polyarthritis, interstitial pneumopathy, Raynaud's phenomenon, “mechanic's hands” skin lesions, and positive anti-aminoacyl-transfer RNA (tRNA) synthetase antibodies. Here, we present seven cases from Gabon.

**Materials and methods:**

This retrospective descriptive study was conducted in the Internal Medicine Department at Libreville University Hospital from January 1, 1984, to December 31, 2023. The study aimed to identify all patients with documented AS and clarify its characteristics.

**Results:**

Seven female patients with a mean age of 42.5 years were identified. The cardinal signs of AS were predominantly muscular (n = 7) and pulmonary (n = 5) with diffuse interstitial pneumopathy (n = 5). The patients experienced stage 2 (n = 3) to stage 3 (n = 2) dyspnea and cutaneous manifestations, including “mechanic's hands” (n = 4). Two patients were positive for Jo-1 antibodies, one for PL7 antibodies, and four for PL12 antibodies.

**Discussion:**

AS poses a significant challenge to clinicians in sub-Saharan Africa due to the diffuse interstitial pneumonitis that accompanies pulmonary involvement.

**Conclusion:**

Our study confirms the predominance of anti-PL12 antibodies and the late diagnosis of this condition.

## Introduction

Le syndrome des antisynthétases (SAS) est une myopathie inflammatoire décrite en 1990 par Marguerie *et al.* [13], qui associe polyarthrite, pneumopathie interstitielle diffuse (PID), syndrome de Raynaud, « mains de mécaniciens », fièvre, et positivité d'autoanticorps anti-aminoacyl-transfer RNA (tRNA) synthétase. Le premier anticorps antisynthétase Jo-1 a été découvert en 1980 chez un patient du nom de John P. présentant une polymyosite couplée à une pneumopathie interstitielle, et dont les deux premières lettres du prénom ont servi de dénomination à cet anticorps. À ce jour, 8 anticorps antisynthétases ont été identifiés : anticorps anti-Jo-1 (histidyl t-RNA), PL7 (thréonyl t-RNA), PL12 (alanine t-RNA), OJ (isoleucil t-RNA) et EJ (glycyl t-RNA), KS (asparagine tR t-RNA NA), anti ZO (phénylalanine t-RNA), et anti HA/YRS (thyrosine t-RNA) [12]. Ce syndrome, comme les maladies auto-immunes en général, semble très peu connu des praticiens d'Afrique subsaharienne et souvent source d’égarement avec certaines maladies infectieuses pulmonaires. Nous rapportons sept observations de SAS dans la population gabonaise dans le but de confirmer l'existence de cette maladie dans ce pays et d'en préciser les particularités.

## Matériel et méthodes

Il s'agit d'une étude monocentrique (service de médecine interne du CHU de Libreville), rétrospective et descriptive, couvrant une période allant du 01/01/1984 au 31/12/2023, et prenant pour support les dossiers de patients hospitalisés dans ledit service, et/ou suivis en ambulatoire. Les patients inclus devaient avoir un diagnostic de SAS documenté (que le diagnostic ait été réalisé en cours d'hospitalisation ou en ambulatoire), et avoir fait l'objet d'un suivi. Les dossiers incomplets ne répondant pas aux précédents critères étaient exclus.

Les variables de l’étude détaillaient les données épidémiologiques, socio-économiques (identité, âge, sexe, profession), cliniques (myosite documentée selon les critères de classification des myopathies inflammatoires révisés par Troyanov *et al.* [18] (Tableau I), avec déficit exprimé selon l’échelle de Chérin) [1], et saisonnières (grande saison sèche de mai à septembre, petite saison sèche de décembre à janvier, grande saison des pluies de février à avril, et petite saison des pluies d'octobre à novembre). Pour le diagnostic de SAS, les patients avaient conjointement bénéficié d'une biopsie musculaire proximale et de la réalisation de dot myosite qui est un panel d'autoanticorps spécifiques de myosites, se faisant en France au laboratoire CERBA.

**Tableau I:** Critères révisés de classification des myopathies inflammatoires proposés par Troyanov ***et al.,*** 2005

1.	Faiblesse musculaire proximale symétrique
2.	Élévation des enzymes musculaires sériques
3.	Modifications myopathiques à l'électromyographie
4.	Infiltration inflammatoire à la biopsie musculaire avec atrophie fasciculaire ou phénomène de dégénérescence ou régénérescence
5.	Présence d'autoanticorps spécifiques des myosites (antisynthétases, anti MI2, ou SRP)
6.	Rash typique de dermatomyosite : rash ou papule de Gottron ou rash héliotrope

Le retentissement du SAS était apprécié sur le plan respiratoire (dyspnée et stadification selon la classification de la *New York Heart Association* (NHYA)), biologique (NFS, C-réactive protéine, numération formule sanguine, sérologie rétrovirale VIH-1, VIH-2), et morphologique (Rx et tomodensitométrie thoracique, épreuves fonctionnelles respiratoires).

Les thérapeutiques utilisées étaient à base de corticoïdes *(per os,* 1 mg/kg/jour de prednisone ou précédée de bolus de méthyl prednisolone), d'immunosuppresseurs (cyclophosphamide intraveineux relayé par du méthotrexate *per os* ou de l'azathioprine *per os).* Leur efficacité était appréciée en fonction de la régression de la dyspnée et de l'atteinte musculaire.

L'efficacité du traitement sur l'atteinte musculaire ainsi que sur la dyspnée était mesurée sur la base de l’échelle de Chérin au moment du diagnostic et à trois mois du traitement immunosuppresseur. Les données évolutives (maintien d'un suivi, perdu de vue, décès), étaient précisées. Toutes les données étaient répertoriées sur une fiche de recueil de données, saisies à l'aide du logiciel Epi Info™. Les données quantitatives étaient décrites à l'aide de moyennes et les données qualitatives à l'aide d'effectifs et de pourcentages.

## Résultats

Sur un total de 83 patients avec myopathies inflammatoires toutes causes confondues, 7 (8,4 %) patientes de 42,5 ans (extrêmes : 26 et 59 ans), avaient un SAS diagnostiqué, majoritairement en saison sèche (n = 5 répartis en 3 en grande saison sèche et 2 en petite saison sèche). Aucun statut socioéconomique ne se dégageait (Tableau II). L'atteinte musculaire (n = 7), l'altération de l’état général (n = 7), l'atteinte respiratoire (n = 5) et cutanée (n = 4) demeuraient au premier plan. Biologiquement la créatine phosphokinase (CPK) était normale dans 5/7 cas. Il existait une hyperleucocytose dans 4/7 cas (moyenne : 13 092; extrêmes : 4 130 et 27 180), une anémie dans 5/7 cas (moyenne : 11,2; extrêmes : 8,9 et 12,8), (Tableau III) avec des plaquettes toujours normales. La sérologie VIH était toujours négative. L’échelle de Chérin moyenne lors du diagnostic de myopathie inflammatoire était de 45/75 (extrêmes : 35 et 70), et le stade de la dyspnée lors de l'hospitalisation de 3 stades 2, 2 stades 3 et 2 stades 0. L'analyse histologique de la biopsie musculaire révélait deux dermatomyosites et cinq polymyosites (dont une polymyosite associée à une sclérodermie). La radiographie thoracique était en faveur d'une PID dans cinq cas, avec à la tomodensitométrie thoracique une fibrose des bases pulmonaires. Les épreuves fonctionnelles respiratoires (n=4) étaient en faveur de syndromes restrictifs (n = 2) et de syndromes mixtes (n = 2), avec dans tous les cas une absence de retentissement cardiaque. Les anticorps antinucléaires étaient positifs dans 3 cas sur 4, et le dot myosite confirmait dans tous les cas une positivité des anticorps antisynthétases, de type Jo-1 (n = 2), PL7 (n = 1), PL12 (n = 4) (Tableau III). Signalons que dans un cas (patiente dont la myosite a été diagnostiquée en 1984), la réalisation du dot myosite s'est faite 10 ans plus tard soit en 1994, lors de l'installation d'une fibrose pulmonaire symptomatique. Sur le plan thérapeutique, la corticothérapie était administrée selon deux modalités : soit sous forme de bolus de méthylprednisolone (n = 3), relayée par de la prednisone à la dose de 1 mg/kg/jour, soit directement par voie orale à la même posologie (n = 4). Ce traitement était couplé à un immunosuppresseur à base de cyclophosphamide administré par voie intraveineuse (n=4), à raison de 500 mg toutes les deux semaines, pour un total de six cures. Un relais était ensuite instauré par méthotrexate (n = 1) ou azathioprine (n = 3). Dans d'autres cas, l'azathioprine était utilisée d'emblée (n = 3) (Tableau IV). L’échelle de Chérin après six cures de cyclophosphamide (couplées à l'adjonction d'une kinésithérapie de renforcement musculaire) retrouvait un score moyen de 70/75 avec restauration de l'autonomie musculaire, et disparition de la dyspnée d'effort dans tous les cas. À la recherche de tumeur bénigne ou maligne pouvant accompagner le SAS, la tomodensitométrie thoraco-abdomino-pelvienne (répétée annuellement) s'est toujours avérée non contributive. Nous déplorons un décès à six mois du diagnostic dans un tableau de détresse respiratoire fébrile. Le maintien d'un suivi a pu être possible pour les patientes restantes.

**Tableau II:** Données épidémiologiques et cliniques

Mois et année de diagnostic	Févr. 1984	Août 2017	Févr. 2019	Août 2019	Avr. 2023	Juin 2023	Nov. 2023
Statut socio-économique	Chômeur	Sœur catéchumène	Agent d'assurance	Néant	Agent du bâtiment	Étudiante	Enseignante
ATCD	Allergies multiples	Néant	Hypothyroïdie post thyroïdectomie	Néant	Trait drépanocytaire (AS)	2 FCS 1er trimestre	Kystectomie
Âge	38 ans	45 ans	53 ans	59 ans	30 ans	26 ans	47 ans
Sexe	F	F	F	F	F	F	F
Saison de diagnostic	PSS	GSS	PSS	GSS	GSP	GSS	PSP
Fièvre	Non	Non	Non	Oui	Oui	Oui	Oui
AEG	Oui	Oui	Oui	Oui	Oui	Oui	Oui
Atteinte musculaire	Myalgies diffuses	Myalgies diffuses	Myalgies diffuses, faiblesse musculaire	Myalgies diffuses	Myalgies diffuses, faiblesse paravertébrale Atteinte proximale MS>MI	Faiblesse musculaire à la marche	Myalgies diffuses
Atteinte cutanée		Mains de mécanicien	Raynaud, sclérodactylie, mains de mécanicien	Mains de mécanicien	Signe de la manucure des mains		
Atteinte articulaire	Néant	3^e^, 4^e^, 5^e^ MTP, 3^e^, 4^e^, 5^e^ orteils	Arthralgie distale des membres	Oui	Coudes, genoux, IPP	Néant	Néant
Atteinte neurologique	Néant	Néant	Fausses routes	Néant	Fausses routes	Néant	Néant
Atteinte respiratoire	Dyspnée stade 2 (NYHA)	Dyspnée stade 3 (NYHA)	Dyspnée stade 2 (NYHA)	Dyspnée stade 3 (NYHA)	Néant	Néant	Dyspnée stade 2 (NYHA)
Signes ORL					Déglutition douloureuse		Dysphagie
Autre			Reflux gastroesophagien	Reflux gastroesophagien			

Légende : ATCD : antécédent; F : féminin; PSS : petite saison sèche; GSS : grande saison sèche; GSP : grande saison des pluies; PSP : petite saison des pluies; AEG : altération de l’état général; MS : membre supérieur; MI : membre inférieur; MTP : métatarso-phalangien; IPP : interphalangiennes proximales

**Tableau III:** Données biologiques, morphologiques, et immunologiques

Mois et année de diagnostic	Févr. 1984	Août 2017	Févr. 2019	Août 2019	Avr. 2023	Juin 2023	Nov. 2023
VS	20	35	49	NR	NR	85/95	NR
CRP	53,4	0,7	4,8	14	NR	10,2	47,5
CPK	Normaux	Normaux	16,5 N	1,4 N	Normaux	Normaux	Normaux
Leucocytes (/mm^3^)	14 300	22 000	4 590	8 420	27 180	4 130	11 030
Hémoglobine (g/dl)	11,2	12,8	11,5	11,2	10,7	12,6	8,9
Plaquettes (/mm^3^)	478000	322000	203000		465000	325000	565000
Taux Gammaglobulinémie	32,g/l polyclonal	14,4 g/l polyclonal			32,g/l polyclonal		
Sérologie VIH 1,2	Négatif	Négatif	Négatif	Négatif	Négatif	Négatif	Négatif
Biopsie musculaire	PM	PM	PM/Sclérodermie	PM	DM	PM	DM
TDM thoracique	Fibrose pulmonaire	Fibrose pulmonaire	Fibrose pulmonaire bibasale	Fibrose pulmonaire	Normale	Normale	Fibrose pulmonaire
EFR	Syndrome mixte	Syndrome mixte	Syndrome restrictif	Syndrome restrictif	NR	NR	NR
Échographie cardiaque	Normale	Normale	Normale	CMD, Dysfonction diastolique grade 2	Normale	Normale	Normale
Dot myosite	Anticorps anti PL12	Anticorps anti PL12	Anticorps anti Jo-1	Anticorps anti P	Anticorps anti PL12	Anticorps anti PL7	Anticorps anti PL12
AAN	Négatif	NR	>1280 centromère	+320 centromère		+160 nucléolaire	NR
ANS	SSA+			SSA+, SSB+ Scl 70+, Jo-1+		Négatif	
ANCA		+80 cytoplasmique de type C-ANCA	Négatif				

Légende : VS : vitesse de sédimentation; NR : non réalisé; CRP : C réactive protéine; CPK : créatine phosphokinase; PM : polymyosite; DM : dermatomyosite; TDM : tomodensitométrie; EFR : épreuves fonctionnelles respiratoires; AAN : anticorps antinucléaires; ANS : anticorps nucléaires solubles; ANCA : anticorps anticytoplasme des polynucléaires neutrophiles

**Tableau IV:** Données thérapeutiques

Mois et année de diagnostic	Févr. 1984	Août 2017	Févr. 2019	Août 2019	Avr. 2023	Juin 2023	Nov. 2023
3 Bolus SMD	Non	Non	Oui	Oui (600 mg/j)	Non	Non	Oui (960 mg/j)
Corticothérapie per os d'emblée	1 mg/kg/j	1 mg/kg/j		1 mg/kg/j	1 mg/kg/j		
Endoxan IV	500 mg/2 semaines soit 6 cures		500 mg/2 semaines soit 6 cures	500 mg/2 semaines soit 6 cures			500 mg/2 semaines soit 6 cures
Imurel per os	Oui	150 mg/j		Oui	150 mg/jour	150 mg/jour	Oui
Méthotrexate per os			15 mg/semaine				
Autres			Trolovol : 1 cp/jour				
			Plaquenil 1cpx2/j				

Légende : SMD : solumédrol; IV : intraveineux : mg/j : milligramme par jour

## Discussion

Le SAS affecterait 25 à 35 % des patients avec myopathies inflammatoires idiopathiques, avec une prédominance féminine. Si la majorité des auteurs s'accordent pour reconnaître un âge diagnostic moyen de survenue se situant autour de 50 ans, certains auteurs retrouvent plutôt un âge compris entre 43 et 60 ans [9]. En effet, dans notre courte série, bien que nous confirmions la prédominance féminine, 5 de nos 7 patientes avaient moins de 50 ans. Les 2 patientes âgées de plus de 50 ans présentaient, quant à elles, un tableau clinique plus complet que les autres. Leur présentation associait une atteinte musculaire, des mains de mécanicien, une atteinte articulaire, une fibrose pulmonaire symptomatique, ainsi qu'une positivité des anticorps anti-Jo-1 et antinucléaires. Dans la littérature d'Afrique subsaharienne, le peu de données existant conforte un âge plus souvent inférieur à 50 ans [8].

Le SAS associe classiquement myopathie inflammatoire, pneumopathie interstitielle, polyarthrite, phénomène de Raynaud, mains de mécanicien, et positivité d'anticorps anti-synthétases. Concernant le diagnostic des myosites, nous avons privilégié les critères des myosites documentées selon les critères de classification des myopathies inflammatoires révisés par Troyanov *et al.* [18] et non ceux de Connors *et al.* [2], qui comportent des critères simples mais non consensuels du SAS : présence d'un anticorps antiARNt-synthétase et de deux critères mineurs (fièvre prolongée inexpliquée, phénomène de Raynaud et mains de mécanicien) ou d'un critère majeur (arthralgies périphériques symétriques, PID ou myosite confirmée [2].

**Figure 1:**
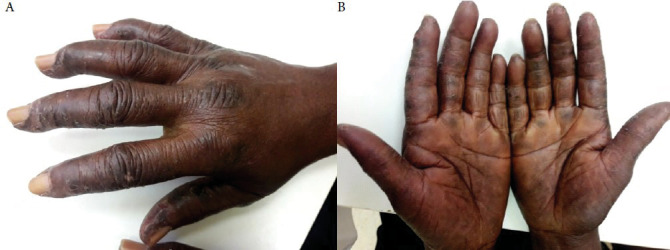
A. Main de mécanicien droite de face (Patient n°7). B. Paumes des mains de mécanicien (Patient n°7)

**Figure 2:**
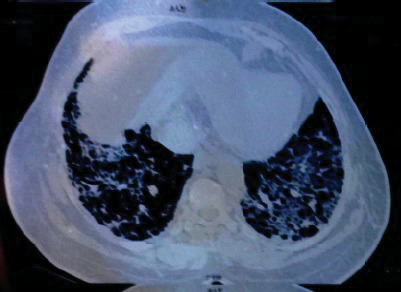
Pneumopathie infiltrative bilatérale et basale d'évolution chronique (Patient n°7)

L'atteinte musculaire était cliniquement présente chez toutes nos patientes avec cependant absence de myolyse chez 5/7 patientes sans remise en cause du diagnostic de myopathie inflammatoire établi par la biopsie musculaire. Cette absence de myolyse peut se retrouver lors d'atrophie musculaire disséminée et ce malgré une myosite chronique active. C'est dans ce cas que le dosage de l'aldolase et de la lacticodéshydrogénase trouve tout son sens, attestant du degré de souffrance musculaire. Nos myopathies inflammatoires étaient plus fréquemment diagnostiquées en saison sèche (5/7 cas) qui correspond aux plus basses températures de l'année. Iba ba *et al.,* dans une étude sur le lupus dans le même pays, retrouvaient également des poussées de lupus plus importantes en saison sèche (petite et grande saison confondues) [7]. La main de mécanicien (ou hyperkératose fissuraire des paumes, des pulpes et des faces latérales des doigts survenant en l'absence de traumatisme et de produits caustiques) (Fig. 1A et 1B) est retrouvée chez 4/7 de nos patientes. Dans la littérature, elle est présente au cours du SAS dans 70 % des cas [5]. Son atteinte est souvent bilatérale et symétrique, présente au moment du diagnostic ou durant l’évolution, considérée par certains auteurs comme un marqueur de l'atteinte viscérale du SAS [15]. Elle est caractéristique du syndrome des antisynthétases, et sa fréquence est variable selon les séries de 21 à 71 % [11, 16]. La PID est retrouvée chez cinq patientes au stade de fibrose (Fig. 2), pouvant évoquer un diagnostic tardif de cette affection, comme rapporté par certains auteurs [7]. Les complications pulmonaires surviennent habituellement chez 5 à 45 % des patients avec SAS [3, 14]. Trois anticorps antisynthétases sont plus fréquemment retrouvés au cours de l'atteinte pulmonaire du SAS, à savoir anti Jo-1, PL7, et PL12. Leur présence impose de réaliser un bilan complémentaire comprenant tomodensitométrie pulmonaire, épreuves fonctionnelles respiratoires (EFR), test de marche de 6 minutes, et échographie cardiaque. Cette atteinte pulmonaire constitue un facteur majeur de morbidité surtout lorsque sont présents des anticorps anti PL7 et anti PL12 [6]. Il a été démontré l'existence dans le tissu pulmonaire de sujets sains de l'autoantigène Jo-1 localisé dans la membrane alvéolaire, suggérant qu'en cas d'immunisation anti-Jo-1 dans une myosite, la réponse immunitaire puisse agresser le poumon [10]. L'existence d'une PID au cours du SAS, impose, en phase aiguë, d'exclure en Afrique subsaharienne plusieurs diagnostics différentiels. Miliaire tuberculeuse, pneumopathie à Covid-19, à *Legionella,* à mycoplasme, à *Chlamydiae,* pneumopathie tuberculeuse, et pneumocystose, doivent plus particulièrement être évoquées. Au cours de la pneumopathie interstitielle chronique, il faut évoquer sarcoïdose, PID associées aux maladies de système, pneumopathie d'hypersensibilité, PID post-radique, PID médicamenteuses, et silicose [4]. La présence d'un seul cas de syndrome de Raynaud et de 2 cas d'atteinte articulaire dans notre série ne nous permet pas de conclure.

Les anticorps antisynthétases agissent contre les aminoacyl-ARNt synthétases. Ces aminoacyl-ARNt synthétases sont des enzymes importantes de la traduction. Elles catalysent la fixation d'un acide aminé sur l'extrémité 2’ ou 3’OH de leurs ARNt synthétases respectifs selon leur classe. La réaction d'aminoacylation se déroule en général en deux étapes et nécessite un acide aminé, l'ARNt, et de l'ATP comme source d’énergie. Leur inhibition par les anticorps anti-amino-acyl-transfer RNA (tRNA) synthétases a pour conséquence l'arrêt de la croissance cellulaire [17]. Dans notre série, nous individualisons trois anticorps antisynthétases qui sont dans la littérature plus fréquemment retrouvés : Jo-1 (2 cas), PL7 (1 cas), et PL12 (4 cas). Les anticorps antinucléaires ont une faible sensibilité car ils peuvent être rendus négatifs dans près de 70 % des cas de SAS sans pour autant exclure ce diagnostic. De ce fait en cas de suspicion diagnostique de SAS, il semble plus utile de réaliser un dot myosite qui combine les anticorps spécifiques des myosites que le dosage des autoanticorps antinucléaires [19]. Alors que nous avions tendance dans notre service à faire systématiquement biopsie musculaire et dot myosite, notre pratique actuelle va dans le sens de ne réaliser que le dot myosite pour diminuer les coûts diagnostiques.

Sur le plan thérapeutique, les immunosuppresseurs le plus souvent prescrits sont le cyclophosphamide, l'azathioprine, le mycophénolate mofétil, la ciclosporine ou le tacrolimus [20]. Aucune étude n'a montré la supériorité de l'un d'eux par rapport aux autres dans la prise en charge des PID des myopathies inflammatoires idiopathiques. Nous avons opté pour l'association d'une corticothérapie et d'un traitement immunosuppresseur pour lequel notre choix s'est majoritairement orienté vers l'azathioprine plutôt que le méthotrexate, du fait de la toxicité pulmonaire de ce dernier. L'association de la corticothérapie au méthotrexate et à l'azathioprine paraît supérieure comparée à l'utilisation de chaque médication en monothérapie [17].

## Conclusion

Le SAS est une maladie autoimmune systémique encore mal connue des praticiens d'Afrique subsaharienne. Ce sous-groupe de myopathie inflammatoire idiopathique constitue un véritable défi pour le clinicien, avec une atteinte cutanée spécifique (mains de mécanicien) pouvant orienter vers le diagnostic. Le dot myosite constitue un outil immunologique essentiel pour le diagnostic avec, au premier plan, les anticorps anti Jo-1, anti PL7, et anti PL12. L'atteinte pulmonaire qui lui est associée ouvre la porte en Afrique subsaharienne à de nombreux diagnostics différentiels pouvant égarer le praticien et rendre le diagnostic tardif.

## Financement de l'étude

Cette étude n'a reçu aucun financement.

## Contribution des auteurs et autrices

Iba Ba Josaphat : conception et rédaction

Mfoumou Annick Flore : rédaction et relecture

Nseng-Nseng Ondo Ingrid : relecture et collecte de données

Kombila Ulrich Davy : recherche bibliographique, relecture

Boguikouma Jean Bruno : conception, relecture, approbation de la version finale

## Conflits d'intérêts

Aucun conflit d'intérêts n'a été déclaré.
