# Supplementary material for: Antisynthetase syndrome: seven observations from the internal medicine department of the Libreville University Hospital, Gabon
Source: Med Trop Sante Int. 2025 Jun 25;5(2):mtsi.v5i2.2025.711. [Article in French] doi: 10.48327/mtsi.v5i2.2025.711 (PMC12305228; doi:10.48327/mtsi.v5i2.2025.711)
Supplement: Related texts

# Research article: Antisynthetase syndrome: seven observations from the internal medicine department of the Libreville University Hospital, Gabon

## Introduction

Antisynthetases syndrome (AS), first described in 1990 by Marguerie *et al. [13],* is an inflammatory myopathy that combines polyarthritis, diffuse interstitial pneumonitis (DIP), Raynaud's syndrome, “mechanic's hands,” fever, and positivity for anti-aminoacyl-transfer RNA (tRNA) synthetase autoantibodies. The first antisynthetase antibody, Jo-1, was discovered in 1980 in a patient named John P., who had polymyositis coupled with interstitial pneumonitis. This antibody was named after the patient's name. To date, eight antisynthetases antibodies have been identified: anti-Jo-1 (histidyl tRNA), PL7 (threonyl tRNA), PL12 (alanine tRNA), OJ (isoleucyl tRNA), EJ (glycyl tRNA), KS (asparagine tRNA), anti-ZO (phenylalanine tRNA), and anti-HA/YRS (thyrosine tRNA) [12]. Like other autoimmune diseases, AS seems to be poorly understood among practitioners in sub-Saharan Africa and is often confused with infectious lung diseases. Here, we present seven cases of AS in the Gabonese population to confirm the presence of this disease in the country and clarify its characteristics.

## Materials and methods

This is a retrospective, descriptive, single-center study based in the internal medicine department of Libreville University Hospital and covering the period from January 1, 1984, to December 31, 2023. The study is based on the records of patients who were hospitalized and/or followed up on an outpatient basis in the department. Included patients had to have a documented AS diagnosis and have been followed up on, whether the diagnosis was made during hospitalization or on an outpatient basis. Incomplete records that did not meet these criteria were excluded.

The study variables included epidemiological and socioeconomic data (e.g., identity, age, sex, and occupation), clinical data (e.g., documented myositis according to the revised classification criteria for inflammatory myopathies by Troyanov *et al.* [18] (Table I), with deficiency expressed according to the Chérin scale [1]), and seasonal data (e.g., long dry season from May to September, short dry season from December to January, long rainy season from February to April, and short rainy season from October to November). Patients underwent both a proximal muscle biopsy and dot myositis testing (a panel of autoantibodies specific to myositis) for the AS diagnosis. This testing was carried out in France at the CERBA laboratory.

**Table I:** Revised criteria for the classification of inflammatory myopathies proposed by Troyanov ***et al.,*** 2005

1.	Symmetrical proximal muscle weakness
2.	Elevated serum muscle enzymes (CK, aldolases, ASAT, ALAT, LDH)
3.	Myopathic changes on electromyography
4.	Inflammatory infiltration on muscle biopsy with fascicular atrophy or degeneration or regeneration
5.	Presence of autoantibodies specific to myositis
6.	Rash typical of dermatomyositis: Gottron's rash or papules, or heliotrope rash

We assessed the impact of AS in terms of respiratory function (dyspnea and staging according to the New York Heart Association (NYHA) classification), biological parameters (complete blood count, C-reactive protein, and HIV-1 and HIV-2 serology), and morphology (chest X-ray, computed tomography (CT) scan, and pulmonary function tests).

The therapies used were based on corticosteroids (1 mg/kg/day of prednisone *per os* or after a bolus of methylprednisolone), immunosuppressants (intravenous cyclophosphamide followed by methotrexate or azathioprine *per os).* Their efficacy was assessed based on regression of dyspnea and muscle involvement.

We measured the efficacy of the treatment on muscle involvement and dyspnea using the Chérin scale at diagnosis and three months after immunosuppressive treatment. Progressive data (continued follow-up, lost to follow-up, and death) were specified. All data were recorded on a data collection form and entered using Epi Info^TM ^software. Quantitative data were described using means, and qualitative data were described using numbers and percentages.

## Results

Out of a total of 83 patients with inflammatory myopathies of all causes, 7 (8.4%) female patients aged 42.5 years (range: 26 and 59 years) had a diagnosis of AS, mainly in the dry season (n=5, divided into 3 in the long dry season and 2 in the short dry season). No socioeconomic status emerged (Table II). Muscle involvement (n=7), general deterioration (n=7), respiratory involvement (n=5), and skin involvement (n=4) remained prominent. Biologically, creatine phosphokinase (CPK) was normal in 5/7 cases. Hyperleukocytosis was present in 4/7 cases (mean: 13,092; extremes: 4,130 and 27,180), anemia in 5/7 cases (mean: 11.2; extremes: 8.9 and 12.8) (Table III) with platelets always normal. HIV serology was always negative. The mean Chérin scale at the time of diagnosis of inflammatory myopathy was 45/75 (range: 35 and 70), and the stage of dyspnea at hospitalization was 3 stage 2, 2 stage 3, and 2 stage 0. Histological analysis of muscle biopsy revealed two cases of dermatomyositis and five cases of polymyositis (including one case of polymyositis associated with scleroderma). Chest X-rays were consistent with DIP in 5 cases, with chest CT scans showing pulmonary base fibrosis. Respiratory function tests (n=4) were consistent with restrictive syndromes (n=2) and mixed syndromes (n=2), with no cardiac involvement in any case. Antinuclear antibodies were positive in 3 out of 4 cases, and dot myositis confirmed in all cases the presence of antisynthetase antibodies, of the Jo-1 (n=2), PL7 (n=1), and PL12 (n=4) types (Table III). It should be noted that, in one case, a patient diagnosed with myositis in 1984 was diagnosed AS 10 years later, in 1994, when they developed symptomatic pulmonary fibrosis. Treatment consisted of corticosteroids administered in two ways: as a methylprednisolone bolus (n=3), followed by prednisone at a dose of 1 mg/kg/day, or orally at the same dosage (n=4). This treatment was combined with an intravenous cyclophosphamide-based immunosuppressant at a dose of 500 mg every two weeks for a total of six courses (n=4). This was then followed by methotrexate (n=1) or azathioprine (n=3). In other cases, azathioprine was used from the beginning (Table IV). After six courses of cyclophosphamide combined with muscle-strengthening physiotherapy, the Chérin scale showed a mean score of 70/75, with restoration of muscle autonomy and disappearance of exertional dyspnea in all cases. In searching for benign or malignant tumors that may accompany AS, thoracoabdominal-pelvic computed tomography (repeated annually) was always inconclusive. One patient died six months after diagnosis due to febrile respiratory distress. Follow-up was possible for the remaining patients.

**Table II:** Epidemiological and clinical data

Month and year of diagnosis	Feb 1984	Aug. 2017	Feb. 2019	Aug. 2019	Apr. 2023	Jun 2023	Nov. 2023
Socioeconomic status	Unemployed	Sister catechumen	Insurance agent	None	Construction worker	Student	Teacher
ATCD	Multiple allergies	None	Postthyroidectomy hypothyroidism	None	Sickle cell trait (AS)	FCS 1st trimester	Cystectomy
Age	38 years	45 years	53 years	59 years	30 years	26 years	47 years
Sex	F	F	F	F	F	F	F
Season of diagnosis	SDS	LDS	DS	LDS	LRS	LDS	SRS
Fever	No	No	No	Yes	Yes	Yes	Yes
AGC	Yes	Yes	Yes	Yes	Yes	Yes	Yes
Muscle involvement	Diffuse myalgia	Diffuse myalgia	Diffuse myalgia, muscle weakness	Diffuse myalgia	Diffuse myalgia, paravertebral weakness Proximal involvement MS>MI	Muscle weakness when walking	Diffuse myalgia
Skin involvement		Mechanic's hands	Raynaud's syndrome, sclerodactyly, mechanic's hands	Mechanic's hands	Sign of manicured hands		
Joint involvement	None	3^rd^, 4^th^, 5^th^ MTP, 3^rd^, 4^th^, 5^th^ toes	Distal arthralgia of the limbs	Yes	Elbows, knees, PIP	None	None
Neurological involvement	None	None	Pulmonary aspiration	None	Pulmonary aspiration	None	None
Respiratory involvement	Stage 2 dyspnea (NYHA)	Stage 3 dyspnea (NYHA)	Stage 2 dyspnea (NYHA)	Stage 3 dyspnea (NYHA)	None	None	Stage 2 dyspnea (NYHA)
ENT signs					Painful swallowing		Dysphagia
Other			Gastroesophageal reflux	Gastroesophageal reflux			

Legend: MH: medical history; F: female; SDS: short dry season; LDS: long dry season; LRS: long rainy season; SRS: short rainy season; AGC: altered general condition; UL: upper limb; LL: lower limb; MTP: metatarsophalangeal joint; PIP: proximal interphalangeal joint

**Table III:** Biological, morphological, and immunological data

Month and year of diagnosis	Feb 1984	Aug. 2017	Feb. 2019	Aug. 2019	Apr. 2023	Jun 2023	Nov. 2023
SR	20	35	49	NP	NP	85/95	NP
CRP	53,4	0,7	4,8	14	NP	10,2	47,5
CPK	Normal	Normal	N	N	Normal	Normal	Normal
Leukocytes (/mm^3^)	14 300	22 000	4 590	8 420	27 180	4 130	11 030
Hemoglobin (g/dl)	11,2	12,8	11,5	11,2	10,7	12,6	8,9
Platelets (/mm^3^)	478000	322000	203000		465000	325000	565000
Gammaglobulin level	polyclonal	polyclonal			polyclonal		
HIV serology 1,2	Negative	Negative	Negative	Negative	Negative	Negative	Negative
Muscle biopsy	PM	PM	PM/Sclerodermy	PM	DM	PM	DM
Chest CT scan	Pulmonary fibrosis	Pulmonary fibrosis	Bibasal pulmonary fibrosis	Pulmonary fibrosis	Normal	Normal	Pulmonary fibrosis
PFT	Mixed syndrome	Mixed syndrome	Restrictive syndrome	Restrictive syndrome	NP	NP	NP
Cardiac ultrasound	Normal	Normal	Normal	DCM, Grade 2 diastolic dysfunction	Normal	Normal	Normal
Dot myositis	Anti-PL12 antibodies	Anti-PL12 antibodies	Anti Jo-1/Scl antibodies	Anti Pm/Scl antibodies	Anti-PL12 antibodies	Anti-PL7 antibodies	Anti-PL12 antibodies
ANA	Negative	NP	centromere pattern >1280	centromere pattern +320		nucleolar pattern +160	NP
anti-ENA	SSA+			SSA+, SSB+ Scl 70+, Jo-1+		Negative	
ANCA		C-ANCA cytoplasmic pattern, +80	Negative				

Legend: SR: sedimentation rate; NP: not performed; CRP: C-reactive protein; CPK: creatine phosphokinase; PM: polymyositis; DCM: dilated cardiomyopathy DM: dermatomyositis; CT: computed tomography; PFT: pulmonary function tests; ANA: antinuclear antibodies; anti-ENA: anti–extractable nuclear antigen antibodies; ANCA: antineutrophil cytoplasmic antibodies

**Table IV:** Therapeutic data

Month and year of diagnosis	Feb 1984	Aug. 2017	Feb. 2019	Aug. 2019	Apr. 2023	Jun 2023	Nov. 2023
3 SMD boluses	No	No	Yes	Yes (600 mg/d)	No	No	Yes (960 mg/d)
Initial oral corticosteroid therapy	1 mg/kg/d	1 mg/kg/d		1 mg/kg/d	1 mg/kg/d		
Endoxan IV	500 mg every 2 weeks for a total of 6 cycles		500 mg every 2 weeks for a total of 6 cycles	500 mg every 2 weeks for a total of 6 cycles			500 mg every 2 weeks for a total of 6 cycles
Imurel orally	Yes	150 mg/j		Yes	153 mg/d	153 mg/d	Yes
Methotrexate orally			15 mg/week				
Others			Trolovol: 1 tablet per day				
			Plaquenil: 1 tablet twice daily				

Legend: SMD: Solumedrol; IV: intravenous; mg/d: milligrams per day

## Discussion

AS is thought to affect 25-35% of patients with idiopathic inflammatory myopathies, with a female predominance. While most authors agree that the average age of diagnosis is around 50 years old, some report an age range of 43 to 60 years old [9]. In our small study, we confirmed the female predominance; however, 5 of our 7 patients were under 50 years of age. The 2 patients over 50 had a more complete clinical presentation than the others. They presented with muscle involvement, mechanic's hands, joint involvement, symptomatic pulmonary fibrosis, and positive anti-Jo-1 and antinuclear antibodies. The limited literature from sub-Saharan Africa supports an average age of diagnosis under 50 years old [8].

AS classically combines inflammatory myopathy, interstitial pneumopathy, polyarthritis, Raynaud's phenomenon, mechanic's hands, and positive anti-synthetase antibodies. We have favored the criteria for documented myositis according to the classification criteria for inflammatory myopathies revised by Troyanov *et al.* [18] rather than those of Connors *et al.* [2], which include simple but non-consensus AS criteria: the presence of anti-tRNA synthetase antibodies and two minor criteria (unexplained prolonged fever, Raynaud's phenomenon, or mechanic's hands) or one major criterion (symmetrical peripheral arthralgia, DIP, or confirmed myositis) [2].

All of our patients exhibited clinical signs of muscle involvement, although myolysis was absent in 5 of 7 patients, which did not call into question the diagnosis of inflammatory myopathy established by muscle biopsy. The absence of myolysis can occur alongside disseminated muscle atrophy despite chronic active myositis. Aldolase and lactate dehydrogenase testing is most useful in this case, as it confirms the degree of muscle damage. Our inflammatory myopathies were more frequently diagnosed during the dry season (5 out of 7 cases), which corresponds to the lowest temperatures of the year. Iba-Ba *et al.,* in a study on lupus in the same country, also found more severe lupus flare-ups during the dry season (short and long seasons combined) [7].

Mechanic's hands, also known as fissured hyperkeratosis of the palms, fingertips, and sides of the fingers, occur in the absence of trauma or caustic products (Fig. 1A and 1B). This condition was found in 4 out of 7 of our patients. In the literature, this condition is present in 70% of AS cases [5]. It is often bilateral and symmetrical, present at diagnosis or during the course of the disease. Some authors consider it to be a marker of visceral involvement in AS [15]. Mechanic's hands are characteristic of antisynthetases syndrome, and their frequency varies between 21% and 71%, depending on the study [11, 16]. DIP was found in five patients at the fibrosis stage (Fig. 2), which may suggest a late diagnosis of this condition as reported by some authors [7]. Pulmonary complications occur in 5-45% of patients with AS [3, 14]. The three most common antisynthetases antibodies found in cases of pulmonary involvement in AS are anti-Jo-1, PL7, and PL12. Their presence requires additional testing, including a pulmonary CT scan, pulmonary function tests (PFTs), a six-minute walk test, and a cardiac ultrasound. This pulmonary involvement is a major factor in morbidity, especially when anti-PL7 and anti-PL12 antibodies are present [6]. The Jo-1 autoantigen has been demonstrated in the alveolar membrane of lung tissue from healthy subjects, suggesting that, in the event of anti-Jo-1 immunization in myositis, the immune response may attack the lungs [10]. The presence of DIP in patients with AS requires ruling out several differential diagnoses in sub-Saharan Africa during the acute phase. In particular, tuberculous miliary tuberculosis, COVID-19 pneumonia, *Legionella* pneumonia, mycoplasma pneumonia, *Chlamydiae* pneumonia, and pneumocystosis should be considered. During chronic interstitial pneumopathy, sarcoidosis, DIP associated with systemic diseases, hypersensitivity pneumonitis, post-radiation DIP, drug-induced DIP, and silicosis should be considered [4]. The presence of one case of Raynaud's syndrome and two cases of joint involvement in our study does not allow us to draw any conclusions.

**Figure 1:** A. Main de mécanicien droite de face (Patient n°7). B. Palms of a mechanic's hands (Patient No. 7)

**Figure 2:** Chronic bilateral basal infiltrative pneumopathy (Patient No. 7)

Antisynthetases antibodies target aminoacyl-tRNA synthetases. These enzymes are important in translation. Depending on their class, they catalyze the attachment of an amino acid to the 2’ or 3’ OH end of their respective tRNAs. The aminoacylation reaction generally occurs in two steps and requires an amino acid, tRNA, and ATP as an energy source. Inhibition of these enzymes by antisynthetases antibodies results in the cessation of cell growth [17]. In our study, we identified three antisynthetases antibodies most frequently reported in the literature: Jo-1 (2 cases), PL7 (1 case), and PL12 (4 cases). However, antinuclear antibodies have low sensitivity because they can be negative in nearly 70% of AS cases without ruling out the diagnosis. Therefore, in cases of suspected AS, it is more useful to perform a dot myositis test, which detects myositis-specific antibodies, than to measure antinuclear autoantibodies [19]. Although our department previously performed muscle biopsies and dot myositis tests systematically, we now perform only dot myositis tests to reduce diagnostic costs.

The most commonly prescribed immunosuppressants for treatment are cyclophosphamide, azathioprine, mycophenolate mofetil, cyclosporine, and tacrolimus [20]. No study has demonstrated the superiority of one over the others for managing DIP. We opted for a combination of corticosteroid therapy and immunosuppressive treatment. Azathioprine is our preferred choice over methotrexate due to the latter's pulmonary toxicity. Combining corticosteroid therapy with methotrexate and azathioprine appears superior to using each medication in monotherapy [17].

## Conclusion

AS is a poorly understood systemic autoimmune disease in sub-Saharan Africa. This subgroup of idiopathic inflammatory myopathy poses a real challenge for clinicians and can be diagnosed by specific skin manifestations, such as mechanic's hands. Anti-Jo-1, anti-PL7, and anti-PL12 antibodies are essential immunological tools for diagnosis. Associated pulmonary involvement can lead to numerous differential diagnoses in sub-Saharan Africa that may mislead practitioners and delay diagnosis.

## Funding

This study received no funding.

## Authors’ contributions

Iba Ba Josaphat: design and writing

Mfoumou Annick Flore: writing and proofreading

Nseng-Nseng Ondo Ingrid: proofreading and data collection

Kombila Ulrich Davy: bibliographic research, proofreading

Boguikouma Jean Bruno: design, proofreading, approval of final version

## Conflicts of interest

The authors declare no conflicts of interest.
